# Effects of Habitat Fragmentation on the Population Structure and Genetic Diversity of Erythroneurini in the Typical Karst Rocky Ecosystem, Southwest China

**DOI:** 10.3390/insects13060499

**Published:** 2022-05-26

**Authors:** Xiaoxiao Chen, Jia Jiang, Ni Zhang, Xiao Yang, Yongkuan Chi, Yuehua Song

**Affiliations:** 1 School of Karst Science, Guizhou Normal University, Guiyang 550001, China; chen75595@163.com (X.C.); 19010170402@gznu.edu.cn (J.J.); 20030170035@gznu.edu.cn (N.Z.); 19010170418@gznu.edu.cn (X.Y.); 201907002@gznu.edu.cn (Y.C.); 2 State Engineering Technology Institute for Karst Desertification Control, Guiyang 550001, China

**Keywords:** Erythroneurini, habitat fragmentation, species diversity, genetic diversity

## Abstract

**Simple Summary:**

In this article, based on satellite imagery, field survey collection, and molecular sequencing data, the habitat fragmentation, species diversity, and genetic diversity of Erythroneurine leafhoppers in three typical karst areas (the Shibing Yuntai Mountain Nature Reserve, the Bijie Salaxi Demonstration Zone, and the Zhenfeng-Huajiang Demonstration Zone) in southwest China were explored. The results of the study show that the fragmentation degree of Erythroneurine leafhopper habitat is affected by the degree of rocky desertification in the area, and there is a positive correlation between them. The species diversity and genetic diversity of Erythroneurine leafhoppers in the study area and its various plots are all affected by habitat fragmentation, and there are certain differences in species, quantity, and gene-flow among different fragmented populations. The weaker the degree of habitat fragmentation in the region, the higher the species diversity and genetic diversity. In order to better protect the species diversity and genetic diversity of other organisms such as the Erythroneurine leafhopper species and their host plants, the following measures can be taken based on the background of the study area. (1) Strengthen the control of rocky desertification; (2) Enhance farmers’ awareness of ecological protection; (3) Increase the continuity of suitable habitats; (4) Improve the stability of the ecosystem; and (5) Improve management policies for protected areas.

**Abstract:**

Karst rocky desertification is one of the main causes of habitat fragmentation in Southwest China. Guizhou Province is located in the center of the karst area in southern China and is a typical karst ecological environment fragile area. Many studies have shown that habitat fragmentation is the main factor leading to the loss of biodiversity and species extinction, and it is also one of the important factors that threaten the survival of natural organisms. This study initially explored the habitat fragmentation degree, species diversity, and genetic diversity of leafhoppers in three typical karst areas in Guizhou. The study was combined with the general situation of the study area, understanding the main factors affecting habitat fragmentation, and putting forward reasonable protection suggestions for species resources. Based on satellite imagery, field survey collection, molecular sequencing data, and related index measurement methods, we measured the habitat fragmentation degree, species diversity index, and genetic diversity index of Erythroneurine leafhoppers of Shibing Yuntai Mountain Nature Reserve, Bijie Salaxi Demonstration Zone, Zhenfeng-Huajiang Demonstration Zone. Moreover, we compared the differences in the three study areas, carried out correlation analysis with relevant environmental factors, and discussed the main factors that formed the results. The results of the study show that the species diversity and genetic diversity of Erythroneurine leafhoppers in the study areas are affected by habitat fragmentation, and the weaker the degree of habitat fragmentation in the region, the higher the species diversity and genetic diversity, which is specifically manifested in species, quantity, and gene-flow. Understanding the status of biodiversity in karst areas is conducive to the sustainable development of biological resources. In order to better protect the diversity of such insects and their host plants and other biological diversity, combined with the background of the research area, we propose corresponding protection measures for reference.

## 1. Introduction

In recent years, due to rapid population growth and over-exploitation of natural resources, the rate of fragmentation of animal and plant habitats has intensified, their habitats have been damaged to varying degrees, and many studies have shown that habitat fragmentation is the main factor leading to the loss of biodiversity and species extinction, and it is also one of the important factors that threaten the survival of natural organisms [1,2,3,4]. Habitat fragmentation refers to the process in which large continuous natural habitats are separated by other unsuitable habitats into many smaller habitat patches (fragments) as a result of human activities and natural interference [5,6]. Habitat fragmentation not only affects species richness and population abundance, but it may also affect the gene-flow between populations, leading to inbreeding, reductions in individual viability, and especially threats to the survival of endangered species; for a fragmented population, if there is no or little gene exchange between the various groups, the genetic diversity of the population will be lost faster, and the genetic differentiation between populations will also be greater [7,8]. Therefore, habitat fragmentation has become one of the focuses of international biodiversity conservation research.

At present, the research on habitat fragmentation and biodiversity is mainly focused on vertebrates and plants, and there is little research on invertebrates, and research on insects is still in its infancy. Based on the fragmentation of different habitats, many scholars have analyzed the species richness, diversity index, spatial distribution, diffusion and immigration, viability of animals and plants, etc. The results show that habitat fragmentation not only restricts the migration of species, but also threatens their survival, leading to a decline in species diversity in the region [9,10,11,12,13,14,15]. Scholars such as Zabel, Liu, Inara, and Yang have tested the relationship between insect communities and habitat fragmentation on fragmented patches of different habitats. The results show that habitat fragmentation has a negative impact on the structure of the insect community. In the significantly fragmented and isolated habitats, the species richness and population abundance of insects are reduced [16,17,18,19]. In terms of genetic diversity, many scholars have studied the genetic diversity of animal and plant populations in different degrees of fragmentation. It is found that the gene-flow between populations is blocked by fragmentation, which affects the connectivity between populations and reduces the genetic diversity of populations [20,21,22,23]. As for insect populations, scholars such as Chen Ting, Zhu Yuan, and Jaipal analyzed the genetic differentiation and gene-flow among different geographic groups of insects such as *Spodoptera exigua* (Hubner), *Empoasca vitis* (Gothe), *Amritodus atkinsoni* (Lethierry), etc. This indicates that landscape types on different spatial scales have different effects on genetic differentiation. The gene-flow between species is affected by geographic barriers, and the genetic distance between populations is not related to geographic distance [24,25,26].

For Southwest China, karst rocky desertification is one of the main causes of habitat fragmentation, which is mainly caused by natural and human factors, such as intense karstification, excessive reclamation, excessive grazing, etc. A large area of karst geology and landforms is distributed in the area, and the outcropping area of carbonate rocks is 426,200 km^2^, mainly in the three provinces of Yunnan, Guizhou, and Guangxi [27]. Among them, Guizhou Province (103°36′–109°35′ E, 24°37′–29°13′ N) is located in the center of the karst area in southern China, with the largest surface carbonate distribution area (130,000 km^2^). Due to its unique hydrogeological structure, the ecological environment in the region is geographical heterogeneity, and this is coupled with the strong interference of human activities, serious soil erosion and rocky desertification, high ecological fragility, and high environmental risks, which seriously affect the regional ecological security and economic development [28,29,30]. Moreover, in different karst geomorphological environments, the survival of animals and plants, including their numbers, types, and structures is affected by the environment. In recent years, most of the ecological environment assessments of typical karst areas in Guizhou are based on the macro-scale, such as landscape pattern, land use, vegetation coverage, etc., while the micro-direction is less involved. The research mostly focuses on ecological sensitivity evaluation, rocky desertification disasters, rocky desertification restoration, etc. However, with the development of science and technology, 3S technology (Remote sensing, Geography information systems, Global positioning systems) and molecular technology have gradually increased, which can combine the macro and micro to evaluate the ecological environment of typical karst areas in Guizhou from different perspectives.

Leafhoppers (Hemiptera: Cicadellidae) are small, rich in species, numerous in number, and widely distributed. The number of known species in the world exceeds 22,000 [31], and they mainly occur in forests and grasslands which feed on plants [32,33]. The population dynamics and distribution patterns of leafhoppers are affected by the climate and host plants, due to the high sensitivity of the environment. As an important part of the ecosystem, leafhopper are also one of the important ecological evaluation indicators, plays a certain role in material circulation, energy flow and environmental change monitoring [34]. Therefore, it can be used as a sensitive environmental indicator. However, the current research of this group is mainly focused on phylogeny, hazards and control applications, classifications, and so on [34]. There are few studies on the ecological environment and the relationship between them and their environment, and no relevant research on the impact of habitat fragmentation on the species diversity and genetic diversity structure of leafhoppers has been reported yet.

Based on field investigations, three typical karst areas in Guizhou Province with certain differences in geographical environment (Shibing Yuntai Mountain Nature Reserve, Bijie Salaxi Demonstration Zone and Zhenfeng-Huajiang Demonstration Zone) were selected as the study area. Exploration of the degree of habitat fragmentation, species diversity and genetic diversity of Erythroneurine leafhoppers in the study area were studied using satellite imagery, field surveys, molecular sequencing, and other data. Moreover, we analyzed the main factors affecting the fragmentation of habitats, thus putting forward reasonable protection suggestions for species resources and providing certain data support and reference bases for related research in the future.

## 2. Materials and Methods

### 2.1. Studied Areas

The Shibing Yuntai Mountain Nature Reserve (108°01′36″–108°10′52″ E, 27°13′56″–27°04′51″ N) is located in the upper middle of Wuyang River Basin in Shibing County. The altitude is 486~1869 m, the annual average temperature is 16.4 °C, and the average annual rainfall is 1117 mm. It belongs to the mid-subtropical warm and humid climate. The area is rich in vegetation types, preserved with strong native karst forests [35], and developed the karst landforms of peak cluster gorges in the dolomites. The main vegetation types are evergreen broad-leaved forests, evergreen and deciduous broad-leaved mixed forests, coniferous forests, secondary shrubs, and shrubs. Due to its low human disturbance and diverse habitats, it has nurtured rich biological resources [36]. It is a typical karst forest ecosystem and is a non-slightly rocky desertification area.

The Bijie Salaxi Demonstration Zone (105°34′59″–105°43′06″ E, 25°37′18″–25°42′37″ N) is located in the Liuchong River Basin, Qixingguan District. The altitude is 1600~2000 m, the annual average temperature is about 13 °C, and the average annual rainfall is 863 mm. It belongs to the north subtropical monsoon humid climate [37]. The vegetation in the area is dominated by broad-leaved forests, coniferous forests, and shrubs. Due to the influence of human factors, the land reclamation rate is high, the native vegetation is mostly destroyed, and the vegetation coverage is low. Nowadays, the main distribution is vine-thorn bush [38]. The terrain in this area is relatively fragmented, with mostly peak clusters and depressions. It is a typical karst plateau mountainous ecological environment, and it is a light–moderate rocky desertification area. 

The Zhenfeng-Huajiang Demonstration Zone (105°01′11″–105°08′38″ E, 27°11′09″–27°17′28″ N) is located along the Beipan River in Huajiang Town, Anshun City. The altitude is 370~1473 m, the annual average temperature is about 18.4 °C, and the average annual rainfall is 1100 mm. It belongs to the southern subtropical dry and hot valley climate. The vegetation in the area is dominated by broad-leaved forests, mixed coniferous and broad-leaved forests, and shrubs. The primary vegetation is seriously damaged and is now mainly secondary vegetation, and there are few types of plants. The topography of the river valley is deep, and the topography has large undulations, which is a typical karst plateau canyon ecological environment [39]. Due to strong human disturbance and long-term soil erosion, the base rock in the demonstration area is exposed, with an area of more than 70%, which is a medium–severe rocky desertification area [40].

### 2.2. Sample Plot Establishment

Nine sample plots of 30 × 30 m were set up, a total of 27 sample plots, from May 2019 to October 2019, according to the different vegetation coverage levels in the three study areas, as well as the occurrence environment and living habits of Erythroneurini leafhoppers, and avoiding patches with strong human interference (spots that directly affect the survival of leafhoppers), in the Shibing Yuntai Mountain Nature Reserve, the Bijie Salaxi Demonstration Zone, and the Zhenfeng-Huajiang Demonstration Zone. The sample plots present different habitat fragmentation states and were distributed as evenly as possible in the study area. Detailed information on the characteristics and vegetation of each plot is shown in Table S1.

The Erythroneurini leafhoppers in the plot were sampled by sweep net. Due to the phototaxis of this kind of insects, in order to ensure the rationality of the collected data, we chose to collect data when the weather was clear and conduct systematic surveys on the sample plots once a month. During the collection, we used insect nets (made of white nylon yarn with a diameter of 30 cm) to sweep 100 nets (200 in total) along the two diagonals of the sample plots. The collected specimens were stored in absolute ethanol, and the vegetation type, temperature, humidity, elevation, and other information in the plot were recorded (Table S1). 

### 2.3. Determination of the Degree of Habitat Fragmentation

This study obtained Landset 8 satellite images with a spatial resolution of 30 m, land use maps, and vegetation coverage maps from the Shibing Yuntai Mountain Nature Reserve, the Bijie Salaxi Demonstration Zone, and the Zhenfeng-Huajiang Demonstration Zone in the summer of 2019. According to the occurrence conditions and living environment of Erythroneurini leafhoppers (phytophagous) and the results of field surveys, the land-use types in the three study areas are divided into grassland, woodland, and other land (cultivated land, construction land, water area, etc.). Among them, grassland and woodland are suitable habitats for leafhoppers. ‘Other land’ is an unsuitable habitat. We used ENVI 5.3 software to preprocess and visually interpret the acquired satellite images of the study area (Supervised classification: maximum likelihood method). In ArcGIS 10.2 software, we performed the tool of Spatial Analysis to obtain the essential elements (such as total area, total perimeter and number of patches of each study area, and the area and perimeter of each fragmented patch, etc.) to describe the degree of habitat fragmentation.

In this study, each adjacent patch is classified differently or discontinuously. We used the patch density index (*PD*), the landscape fragmentation index of the entire study area (*FN*_1_), the landscape fragmentation index of a certain landscape patch type (*FN*_2_, such as grassland), the separation index of landscape type *i* (*N_i_*), the fragmentation degree index (*F*), and the fragmentation index of habitat area within landscape type (*FI*_1,_ *FI*_2_) to determine the fragmentation degree of the Erythroneurini leafhopper habitat in the three study areas [41,42,43,44]. The specific calculation formula is as follows.

(1) $$PD=\frac{N}{A}$$

(2) $$FN_{1}=\frac{\left(N-1\right)}{N_{c}},\;FN_{2}=\frac{\left(N_{i}-1\right)}{MPS}$$

(3) $$C_{i}=\frac{D_{i}}{S_{i}},\;D_{i}=\frac{1}{2}\sqrt{\frac{n_{i}}{A}}\;,\;S_{i}=\frac{A_{i}}{A}$$

(4) $$F=1-\sum_{i=1}^{{}_{{}_{n_{i}}}}\frac{1}{B_{i}}\times {\left(\frac{A_{i}}{A}\right)}^{2},\;B_{i}=\frac{P_{i}}{2\times \sqrt{\pi -A_{i}}}$$

(5) $$FI_{1}=1-\frac{A_{i}}{A},\;FI_{2}=1-\frac{A_{l}}{A}$$

where *A*: Total landscape area, *N*: Total number of patches, *N_c_*: Total area of study area/Minimum patch area, *MPS*: The average patch area of the entire landscape, *N_i_*: The number of patches of landscape type i, *D_i_*: Distance index of landscape type i, *S_i_*: Area index of landscape type i, *A_i_*: The total area inside a certain landscape type, *B_i_*: Plaque shape index, *P_i_*: Plaque circumference, and *A_l_*: The largest patch of this landscape type. The larger the *PD* value, the higher the degree of fragmentation in the study area; *FN*_1_ and *FN*_2_ range between 0 and 1, 1 means that the landscape is completely destroyed, and 0 means that the landscape is not destroyed at all; the greater the degree of separation *C_i_*, the more discrete the plaques and the greater the distance between them; the larger the *F* value, the higher the degree of fragmentation in the study area and the worse the habitat quality; the values of *FI*_1_ and *FI*_2_ are positively correlated with the degree of fragmentation.

### 2.4. Species Diversity Analysis

In the laboratory experiment, the collected samples were classified, dissected, identified, and counted. Erythroneurine leafhopper male specimens were selected to observe their appearance and morphological characteristics. Then, we removed the abdomens and soak them in 5~10% NaOH solution for for 8–12 h. After washing with water, anatomy studies were performed under an Olympus stereoscope to observe the characteristics of the genitals and store them in glycerin, according to the 3I (3I Interactive Keys and Taxonomic Databases) taxonomic database and leafhopper-taxonomy-related literature for species identification [45,46], and using Excel software to establish a database.

With reference to relevant research methods and statistical data of the Erythroneurini leafhopper [47,48,49,50], the differences in the composition and distribution of the Erythroneurini leafhopper community were compared and analyzed in different habitat fragmentations. Furthermore, the relative abundance (*Ra*) of the group’s dominance was calculated [47]. We used the Margalef species richness index (*R*), the Shannon–Wiener diversity index (*H′*), Simpson dominance index (*C*), and Pieluo uniformity index (*J′*) to represent the diversity of the Erythroneurini leafhopper community structure [48,49,50]. The specific calculation formula is as follows. In addition, we used R 3.6.1 soft to conduct significant difference analysis on the number of individuals, genera, and species, as well as each biodiversity index.

(6) $$Ra=(N_{i}/N)100\%$$

(7) R=(S−1)/InN

(8) $$H^{\prime }=-\sum_{i=1}^{S}P_{i}InP_{i},\;P_{i}=N_{i}/N$$

(9) $$C=\sum_{i=1}^{S}\left(N_{i}/N\right)$$

(10) $$J^{\prime }=H^{\prime }/InS$$

where *N_i_*: Number of individuals of the species i, *N*: Total number of individuals, *S*: Total number of species. *Ra* defines rare taxa as <1%, common taxa from 1% to 5%, and dominant taxa as >5%. The higher the *R* value, the higher the richness of the species. The higher the value of *H′*, the higher the complexity of the community and the higher the species diversity. The larger the *C* value, the higher the concentration of the community, the more uneven the distribution of the number of individuals of different species, and the more prominent the ecological functions of the dominant species. The larger the *J′*, the more even the distribution among species, which means that there are fewer rare and dominant species.

### 2.5. Genetic Diversity Analysis

We collected the Erythroneurini leafhopper specimens from different locations from May 2019 to October 2019; they were immersed in absolute ethanol, labeled, and stored in a refrigerator at −20 °C for later use. Based on the results of habitat fragmentation and the type of statistical results of Erythroneurini leafhopper species, we selected *Empoascanara sipra*, which is distributed in the three study areas, to determine the mitochondrial genome fragments *Cytb*, *16S* rRNA and *COI* of nine different geographic populations. Detailed information on the characteristics and vegetation of each plot is shown in Table S2.

The heads and chests of the Erythroneurini leafhoppers were ground and extracted according to the instructions of the DNA extraction kit, and the extracted DNA was stored in a refrigerator at –20 °C for later use. Based on the existing literature and the mitochondrial genome sequence of the Typhlocybinae insects from the National Center for Biotechnology Information (NCBI), a primer design was carried out using the software Primer Premier 6, and the obtained DNA (Extraction kit, TIANGEN BIOTECH) was amplified by PCR and sent to relevant companies (Sangon Biotech (Shanghai) Co., Ltd, No. 698, Xiangmin Road, Songjiang District, Shanghai, China) for sequencing. Refer to Table S3 for specific amplification primer information, refer to Table S4 for specific data of the reaction system, and Table S5 for PCR reaction conditions. Accession numbers are MW821357, MW821364, MW821384~MW821390, MW824347~MW824355, and MW821392~MW821400.

Sequencing data was assembled using DNAStar software, and a homology search was performed through the Blast function in NCBI to determine the amplified sequence as the target sequence to ensure the accuracy of the sequencing results. Analyses of the nucleotide diversity, average nucleic acid difference, and haplotype diversity in the fragmented populations of Erythroneurini leafhoppers were conducted through MEGA 5.0 software and Dna SP 5.10 software, and the *F_st_* value and gene-flow (*N_m_*) between the populations [42], *N_m_* = (1/*F_st_* − 1)/2 (*F_st_*: allele frequency) were calculated.

### 2.6. Correlation Analysis

RDA analysis was performed using Canoco 5.0 software to detect the impact of environmental factors on biodiversity, and Pearson correlation analysis was performed using the R language.

## 3. Results and Analysis

### 3.1. Determination of the Degree of Habitat Fragmentation

#### 3.1.1. Habitat Spatial Distribution of Erythroneurine Leafhoppers in the Study Area

**Figure 1:**
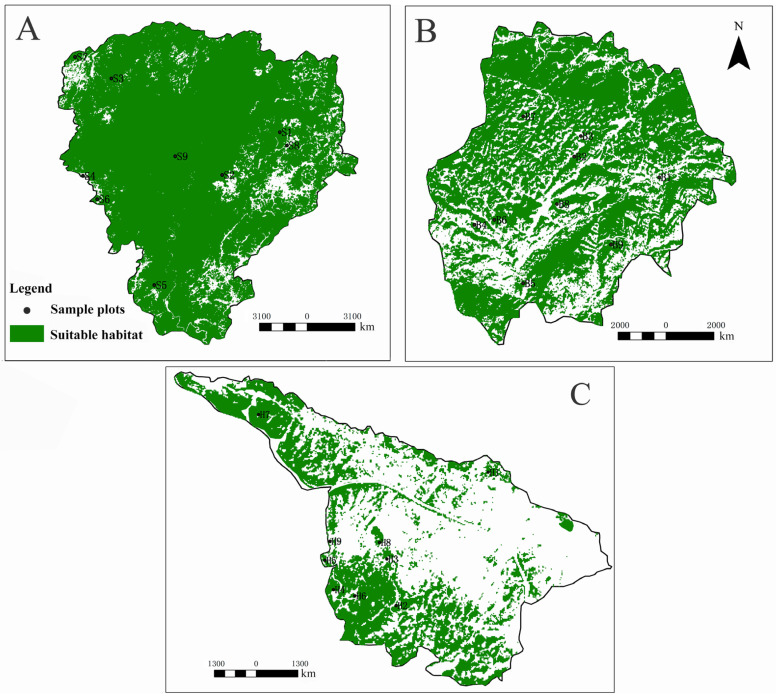
Spatial distribution of habitat of Erythroneurine leafhoppers in study areas. ((**A**): Shibing; (**B**): Bijie; (**C**): Huajiang).

From Figure 1, it can be seen that there are differences in the habitat conditions of the leafhoppers in the demonstration areas of the Shibing Yuntai Mountain Nature Reserve, the Bijie Salaxi Demonstration Zone, and the Zhenfeng-Huajiang Demonstration Zone, and the habitat distributions of leafhoppers are also different. Compared with the other research areas, the Shibing Yuntai Mountain Nature Reserve had the highest internal habitat continuity and the lowest degree of habitat fragmentation. Most areas have vegetation, and suitable habitats account for 91.06% of the total area, while unsuitable habitats are mostly distributed at the edge of the reserve and the eastern region. The fragmentation of the Bijie Salaxi Demonstration Zone habitat is the second. The suitable and unsuitable habitats are alternately scattered throughout the area. The suitable habitats account for 68.05% of the total area, and the unsuitable habitats are mostly distributed in the south. The Zhenfeng-Huajiang Demonstration Zone has the highest degree of habitat fragmentation, with suitable habitats only accounting for 35.53% of the total area, mainly in the southwest and northwest, and most of the rest being unsuitable habitats.

#### 3.1.2. Habitat Fragmentation of Three Different Study Areas

**Figure 2:**
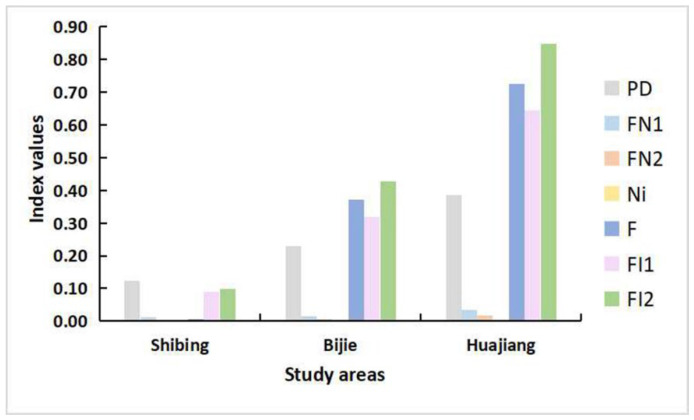
Habitat fragmentation index of Erythroneurine leafhoppers in three different study areas. (*PD*: Patchdensity index; *FN*_1_ and *FN*_2_: Landscape fragmentation index; *Ni*: Separateness index; *F*: Fragmentation index; *FI*_1_ and *FI*_2_: Inside habitat area fragmentation index).

The different habitat fragmentation indexes of leafhoppers are the lowest in the Shibing Yuntai Mountain Nature Reserve, with an average of 0.0537. The Bijie Salaxi Demonstration Zone is the second, with an average fragmentation index of 0.1955. The Zhenfeng-Huajiang Demonstration Zone is greatly affected by human factors and the natural environment, and its habitat fragmentation is the strongest, with an average fragmentation index of 0.3799 (Figure 2 and Table S6).

#### 3.1.3. Habitat Fragmentation of the Sample Plots

**Figure 3:**
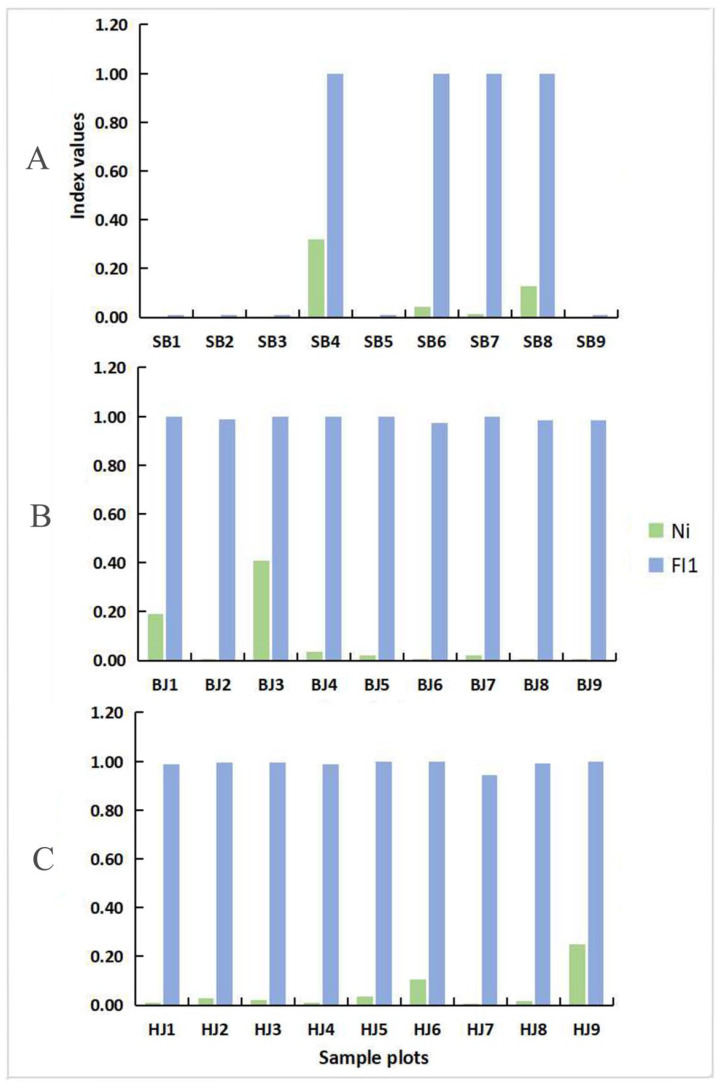
Habitat fragmentation index of Erythroneurine leafhoppers in the sample plots. ((**A**): Shibing; (**B**): Bijie; (**C**): Huajiang; *Ni*: Separateness index; *FI*_1_: Inside habitat area fragmentation index).

The results show (Figure 3 and Table S7) that in the sample plots of Shibing Yuntai Mountain Nature Reserve, SB1, SB2, SB3, SB5, and SB9 have the same degree of habitat fragmentation, strong habitat continuity, and the lowest fragmentation index, with an average of 0.0089. SB4 is located at the edge of the nature reserve, mostly blocked by other land, and has the smallest patch area, with the highest fragmentation index, the average value of which is 0.6674. The fragmentation degree of the remaining plots is ranked from low to high as SB7 < SB6 < SB8. Among the various plots in the Bijie Salaxi Demonstration Zone, BJ6 has the largest habitat continuity area, and its fragmentation index is the lowest, with an average of 0.4916, while BJ3 is the highest, with an average of 0.7471. In addition, the habitat fragmentation degree of the remaining plots is BJ9 < BJ8 < BJ2 < BJ5 < BJ7 < BJ4 < BJ1. Among the various plots in the Zhenfeng-Huajiang Demonstration Zone, the HJ7 plot had a higher vegetation coverage and the lowest fragmentation index, with an average of 0.4914, while the rocks of the HJ9 plot are more exposed and the degree of fragmentation is the highest, with an average of 0.7096. The order of habitat fragmentation in the remaining sample plots is HJ1 < HJ4 < HJ8 < HJ3 < HJ2 < HJ5 < HJ6.

### 3.2. Effects of Habitat Fragmentation on the Species Diversity of Erythroneurine Leafhoppers

#### 3.2.1. Characteristics of Spatial Distribution and Dominance of Community

In the Shibing Yuntai Mountain Nature Reserve, the Bijie Salaxi Demonstration Zone, and the Zhenfeng-Huajiang Demonstration Zone, a total of 7064 Erythroneurine leafhopper specimens were captured, belonging to 51 species in 14 genera. The number of specimens of the Erythroneurine leafhopper was Bijie (5245, 74.4%) > Shi Bing (1580 heads, 22.4%) > Huajiang (239 heads, 3.2%), and the order of the number of units at different levels of the taxa is Shi Bing (10 genera, 25 species) > Bijie (8 genera, 16 species) > Huajiang (8 genera, 14 species).

**Figure 4:**
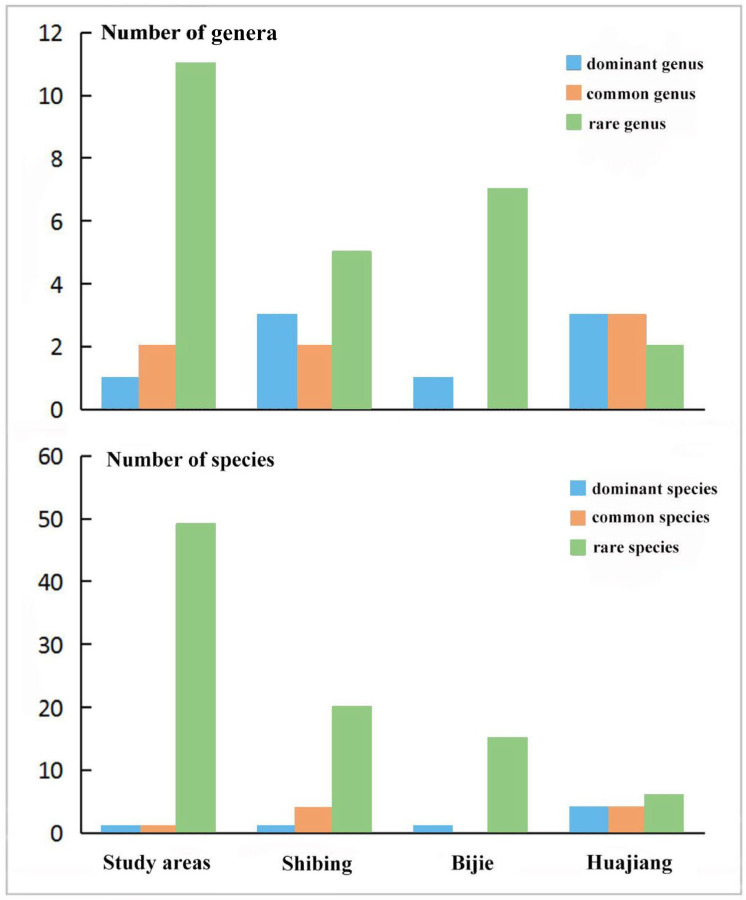
Abundance of genera and species of Erythroneurine leafhoppers in different study areas.

The statistical analysis results show that, in terms of species composition, the proportion of rare genera and species is relatively high in the three study areas, and dominant genera and species are scanty (Table 1 and Figure 4). At the genus level, *Empoascanara* is the dominant taxon in the three study areas, accounting for 91.9% of the total number of individuals; *Mitjaevia* and *Seriana* are common taxa, accounting for 2.3% and 3.6% of the total number of individuals, respectively; the remaining 11 genera are rare taxa, accounting for 2.2% of the total number of individuals, such as *Diomma*, *Thaia*, etc.; among them, *Empoascanara* and *Mitjaevia* are the most abundant species, with 11 and 12 species, respectively. At the species level, *Empoascanara sipra* Dworakowska, 1980 is the dominant taxon in the study area, accounting for 91.5% of the total number of individuals; *Seriana bacilla* Tan, Yuan and Song, 2020 is a common taxon, accounting for 3.4% of the total number of individuals; *Mitjaevia diana* (Distant, 1918), *Empoascanara dwalata* Dworakowska, 1977 and other 47 species are rare taxa, accounting for 5.1% of the total number of individuals.

At the genus level of the Shibing Yuntai Mountain Nature Reserve, *Empoascanara*, *Mitjaevia*, and *Seriana* are the dominant taxa, accounting for 81.3%, 8.2% and 6.3% of the number of individuals, respectively; *Arboridia* and *Salka* are common taxa, accounting for 1.7% and 1.2% of the number of individuals, respectively; the remaining five genera such as *Ziczacella* are all rare taxa, accounting for 1.3% of the individuals. Among them, *Empoascanara* and *Mitjaevia* have the most abundant species, with seven and six species, respectively. At species level, *Empoascanara sipra* Dworakowska, 1980 is the dominant taxa, accounting for 80.2% of the number of individuals; *Mitjaevia protuberanta* Song, Li and Xiong, 2011, *Mitjaevia shibingensis* Chen, Song and Webb, 2020, *Salka sawna* Song and Li, 2011, *Seriana bacilla* Tan, Yuan and Song, 2020 are common taxa, accounting for 15.2% of the number of individuals; *Empoascanara gracilis* Dworakowska, 1992, *Mitjaevia aurantiaca* (Mitjaev, 1969) and other 18 species are rare taxa, accounting for 4.6% of the number of individuals.

At the genus level of the Bijie Salaxi Demonstration Zone, *Empoascanara* is the dominant taxon, accounting for 98.9% of the number of individuals; the remaining seven genera such as *Elbelus* and *Tautoneura* are all rare taxa, accounting for 1.1% of the number of individuals; among them, *Kapsa* and *Mitjaevia* have the most species, with three and five species, respectively. In terms of the species level, *Empoascanara sipra* Dworakowska, 1980 is the dominant taxon, accounting for 98.9% of the number of individuals; the 15 species such as *Kapsa arca* Song and Li, 2008, *Tautoneura albida* Dworakowska, 1970, etc., are rare taxa, accounting for 1.1% of the individuals. The dominant species in the area are very prominent, and there are no common genera and species distribution.

In the genus level of the Zhenfeng-Huajiang Demonstration Zone, *Elbelus*, *Empoascanara*, and *Seriana* are dominant taxa, accounting for 18.5%, 7.9%, and 64.5% of the number of individuals, respectively; *Kapsa*, *Thaia*, and Erythroneurini new-genu-2 are common taxa, accounting for 2.9%, 1.7%, and 3.3% of the number of individuals, respectively; *Arboridia* and *Mitjaevia* are rare taxa, accounting for 0.8% and 0.4% of the number of individuals, respectively; among them, *Empoascanara* is the most diverse species, with a total of four species. At species level, *Empoascanara sipra* Dworakowska, 1980, *Seriana bacilla* Tan, Yuan and Song, 2020, *Seriana ochrata* Dworakowska, 1971, and *Elbelus tripunctatus* Mahmood, 1967 are the dominant taxa, accounting for 88.3% of the number of individuals; Erythroneurini new-2.sp. nov, *Empoascanara* sp.fm-6, *Kapsa* sp.nov-1, *Thaia* sp.fm-2 are common taxa, accounting for 8.4% of the number of individuals; *Empoascanara mai* Dworakowska, 1992, *Kapsa dolka* Dworakowska, 1979 and other four species are rare taxa, accounting for 3.3% of the number of individuals.

#### 3.2.2. Distribution Differences of Community Composition 

**Figure 5:**
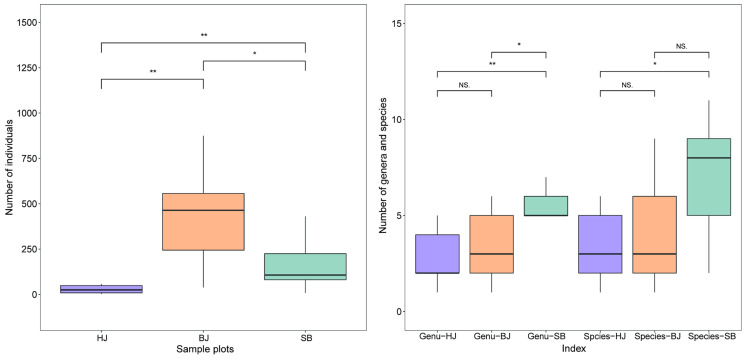
The number of individuals, geners, and species of Erythroneurine leafhoppers in different study areas and plots. (*, *p* < 0.05; **, *p* < 0.01). NS: No significant.

According to the data in Table S12, we made a significantly different analysis of the species diversity in the three study areas. The results show that there were significant differences in the number of individuals (*p* = 0.0018, 0.008, 0.043), genera (*p* = 0.0091, 0.043, 0.68), and species (*p* = 0.032, 0.12, 0.65) of Erythroneurine leafhoppers in the three study areas with different degrees of habitat fragmentation (Figure 5). The specific species composition and abundance are shown in Tables S8–S12. In terms of the number of individuals, the Bijie Salaxi Demonstration Zone has the most, and the Zhenfeng-Huajiang Demonstration Zone has the least. The genus-level and species-level results were the same, with significant differences in the number of species, except between Huajiang and Bijie. In addition, the Shibing Yuntai Mountain Nature Reserve has the most abundant species and Huajiang the least. Combined with the fragmentation situation in the study area, the results indicate that the community composition of leafhoppers was affected by the different environments, and habitat fragmentation may be one of the important reasons.

#### 3.2.3. Diversity of Spatial Distribution of Erythroneurine Community

**Figure 6:**
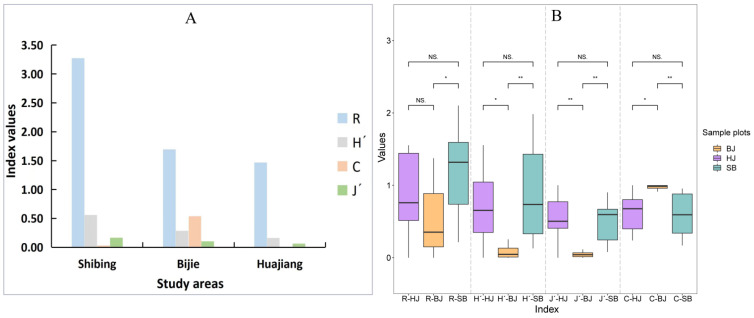
Community diversity of Erythroneurine leafhoppers in three different study areas ((**A**): the total values of three study areas. (**B**): the values of nine sample plots of three study areas). *, *p* < 0.05; **, *p* < 0.01. *R*, Margalef Species richness index; *H′*, Shannon–Wiener Diversity index; *C*, Simpson Dominance index; *J′*, Pieluo Uniformity index.

Based on the statistics of the number of species and individuals (Table S12), we calculated the species diversity indicators of leafhoppers in the three study areas. The results show that there are differences among the three study areas (p*_R_* = 0.034~0.22, p*_H′_* = 0.034~0.22, p*_J′_* = 0.002~0.6, p*_C_* = 0.002~0.86), especially the Bijie Salaxi Demonstration Zone (Figure 6 and Table S11). The spatial distribution of the species richness index *R*, the diversity index *H′*, and the uniformity index *J′* in the three study areas are Shibing > Bijie > Huajiang, and the dominance index *C* is Bijie > Shibing > Huajiang. The Erythroneurine leafhoppers community composition is the most abundant in the Shibing Yuntai Mountain Nature Reserve, and the number of various groups is relatively uniform; its species diversity index is the highest, with an average of 1.0069. The difference in species diversity index between the Bijie Salaxi Demonstration Zone and the Zhenfeng-Huajiang Demonstration Zone is small. Apart from the dominance index C, the other indexes are slightly higher than the latter, and the average species diversity indexes are 0.6550 and 0.4228, respectively. The dominance index of Bijie is more prominent since the number of individuals of *Empoascanara sipra* collected in Bijie was much higher than that of the other two study areas. Although *Empoascanara sipra* as a widespread species has been found in many areas of Guizhou, it prefers to live on grass and shrubs, such as water hemp. There are many types of grass and shrub vegetation in the Bijie Salaxi Demonstration Zone, while the vegetation structure of the Shibing Yuntaishan Nature Reserve is dominated by arbor, and the types and numbers of vegetation in Huajiang are relatively small. The suitable habitat for Erythroneurine leafhoppers is more common in the Bijie, so the number of individuals there is the most abundant.

**Figure 7:**
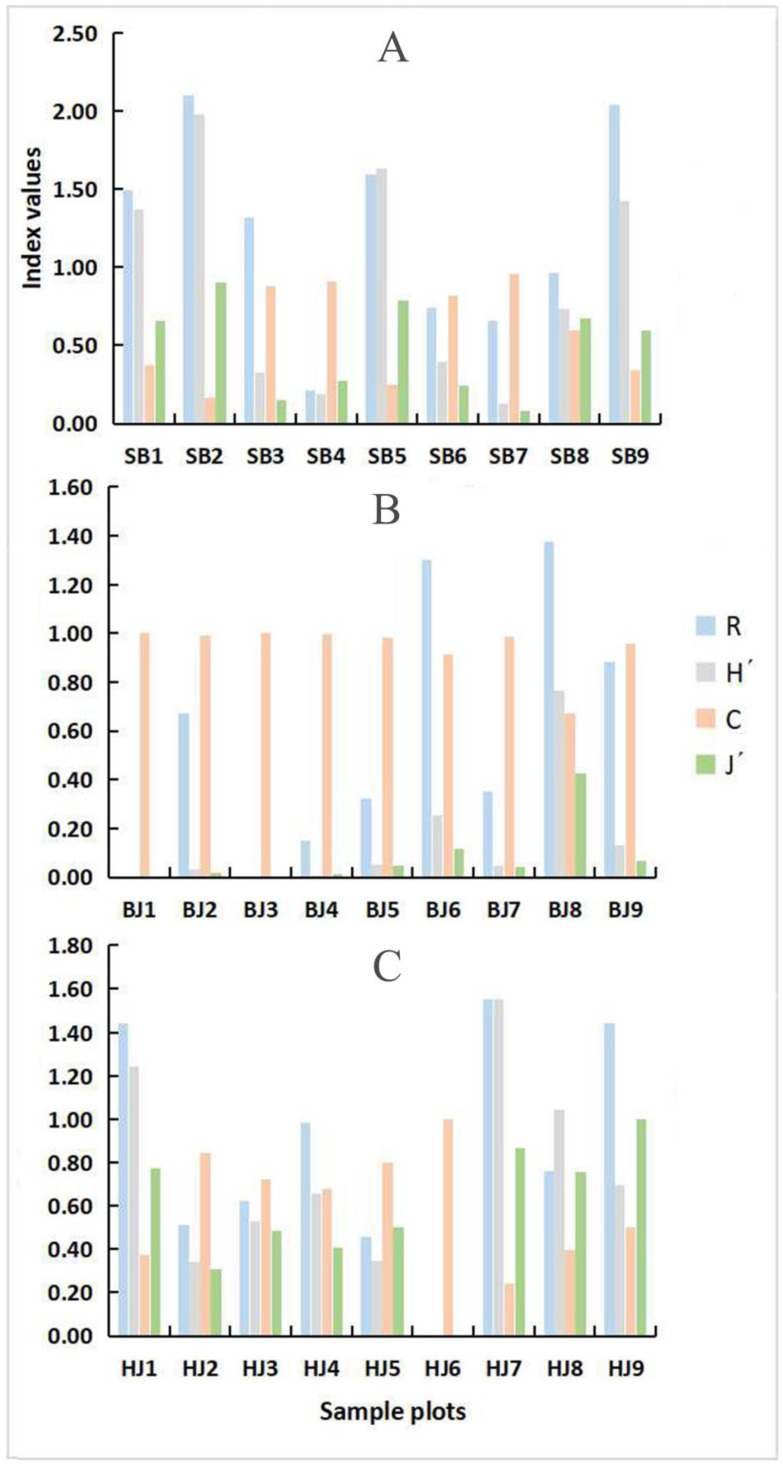
Community diversity of Erythroneurine leafhoppers in each sample plot ((**A**): Shibing; (**B**): Bijie; (**C**): Huajiang). *R*, Margalef Species richness index; *H′*, Shannon-Wiener Diversity index; *C*, Simpson Dominance index; *J′*, Pieluo Uniformity index.

The specimens of Erythroneurine leafhoppers collected in each plot in the three study areas were classified, identified, and counted, and the community diversity characteristics of this tribe in different habitat fragmentation environments were analyzed. The specific research results are shown in Figure 7 and Table S12. 

In the Shibing Yuntai Mountain Nature Reserve, according to the calculation result of diversity index, the spatial distribution of the species richness index *R* is SB2 > SB9 > SB5 > SB1 > SB3 > SB8 > SB6 > SB7 > SB4, the diversity index *H′* is SB2 > SB9 > SB5 > SB1 > SB8 > SB6 > SB3 > SB7 > SB4, the dominance index *C* is SB7 > SB4 > SB8 > SB6 > SB3 > SB1 > SB9 > SB5 > SB2, and the uniformity index *J′* is SB2 > SB5 > SB8 > SB1 > SB9 > SB4 > SB6 > SB3 > SB7. The species diversity of leafhoppers approached in the SB1, SB2, SB5, and SB9 plots, because the habitat fragmentation degree of these four plots was consistent and the lowest. Therefore, the species were evenly distributed, the community composition was relatively rich, and the diversity index was high, with an average value between 0.9755 and 1.2890. Among the remaining sample plots, SB4 was severely blocked by unsuitable habitats due to its location at the edge of the reserve, with the smallest patch area, the highest degree of habitat fragmentation, single community structure, and the lowest diversity index, with an average of 0.3979.

In the Bijie Salaxi Demonstration Zone, the spatial distribution of the species richness index *R* is BJ8 > BJ6 > BJ9 > BJ2 > BJ7 > BJ5 > BJ4 > BJ3 > BJ1, the diversity index *H′* is BJ8 > BJ6 > BJ9 > BJ5 > BJ7 > BJ2 > BJ4 > BJ3 > BJ1, the dominance index *C* is BJ3 > BJ1 > BJ4 > BJ2 > BJ7 > BJ5 > BJ9 > BJ6 > BJ8, and the uniformity index *J′* is BJ8 > BJ6 > BJ9 > BJ5 > BJ7 > BJ2 > BJ4 > BJ3 > BJ1. Sample plots such as BJ6, BJ8, and BJ9 have large patch areas, so the degree of habitat fragmentation is low, and the species diversity index of Erythroneurine leafhoppers is relatively high; among them, BJ6 is the highest with an average value of 0.6455. The number and types of species in this plot are relatively higher, and the distribution is more uniform. In the BJ1, BJ3, and BJ4 plots, the habitat fragmentation is high, and the vegetation coverage is less, which is not conducive to survival and prevents the migration of Erythroneurine leafhoppers, so the species diversity is reduced, with an average value of 0.2500.

In the Zhenfeng-Huajiang Demonstration Zone, the spatial distribution of the species richness index *R* is HJ7 > HJ9 > HJ1 > HJ4 > HJ8 > HJ3 > HJ2 > HJ5 > HJ6, the diversity index *H′* is HJ7 > HJ1 > HJ8 > HJ9 > HJ4 > HJ3 > HJ5 > HJ2 > HJ6, the dominance index *C* is HJ6 > HJ2 > HJ5 > HJ3 > HJ4 > HJ9 > HJ8 > HJ1 > HJ7, and the uniformity index *J′* is HJ9 > HJ7 > HJ1 > HJ8 > HJ5 > HJ3 > HJ4 > HJ2 > HJ6. The degree of habitat fragmentation is low in those sample plots, such as HJ1, HJ4, and HJ7, etc., with a relatively rich community structure and high diversity index; among them, HJ7 is the highest, with an average of 1.0536. The HJ2, HJ5, and HJ6 plots have a relatively high degree of habitat fragmentation, with fewer species, prominent dominant populations, and low distribution uniformity; among them, HJ6 has the lowest diversity index, with an average of 0.2500.

### 3.3. The Impact of Habitat Fragmentation on Population Gene-Flow

The mitochondrial genome fragments *Cytb* (59bp), *16S* rRNA (485bp), and *COI* (671bp) of *Empoascanara sipra* were obtained by sequencing. The results of the study indicate that there are certain genetic differences among the populations of different areas of habitat fragmentation. The Shibing Yuntai Mountain Nature Reserve has the lowest degree of habitat fragmentation; its population of Erythroneurine leafhoppers has the richest genetic diversity, and each nucleotide polymorphism parameter value is the highest. The Bijie Salaxi Demonstration Zone is second, and the Zhenfeng-Huajiang Demonstration Zone has the most severe degree of habitat fragmentation, and the genetic diversity of population is the lowest. The statistics of each genetic difference parameter are shown in Table 2.

**Figure 8:**
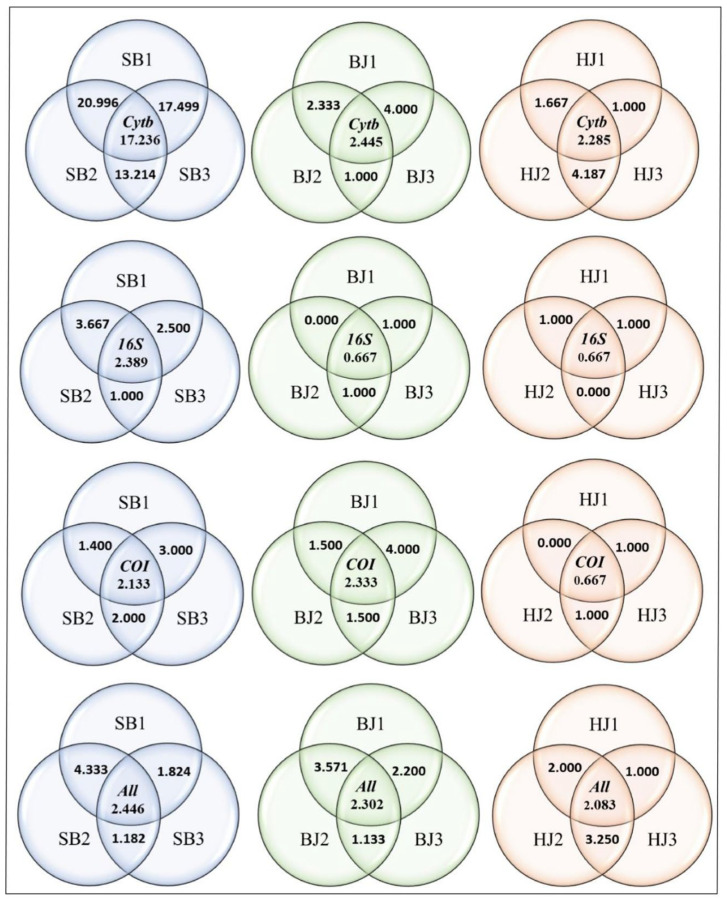
Gene-flow (*N_m_*) of fragmented populations of Erythroneurine leafhoppers in the study areas.

From Table S13 and Figure 8, it can be seen that the *F_st_* values among the partially fragmented populations in the three study areas are all ≤0, which indicates that gene exchanges between fragmented populations are common. In order to facilitate the comparison of the gene-flow of the fragmented populations in the three protected areas, the *Gamma_st_* value was used instead of the *F_st_* value to estimate the gene-flow, and the gene-flow of the fragmented populations of Erythroneurine leafhoppers in the protected area was obtained.

The gene-flow (*N_m_*) of the fragmented population of *Empoascanara sipra* in the Shibing Yuntai Mountain Nature Reserve is greater than that of the other two study areas, the Bijie Salaxi Demonstration Zone is second, and the Zhenfeng-Huajiang Demonstration Zone is the smallest, with an average of *N_m SB_* (6.0511) > *N_m BJ_* (1.9365) > *N_m HJ_* (1.4254). The average *F_st_* of the fragmented population in Shibing is −0.3834, the gene-flow (*N_m_*) is ≥ 1, the maximum is 20.9961, and the average *N_m_* of each gene segment is between 2.1333 and 17.2361. The average *F_st_* of the fragmented population in Bijie is −0.3333, and the gene-flow (*N_m_*) is between 0 and 4. The population has no gene exchange in the *16S* rRNA, and the average *N_m_* of each fragment is between 0.6667 and 2.4445. The average *F_st_* of the fragmented population in Huajiang is −0.3333, and the gene-flow (*N_m_*) is between 0 and 4.1870. There is no gene exchange in the *16S* rRNA and *COI* in the population, and the average *N_m_* of each fragment is between 0.6667 and 2.2846. The results show that the habitat fragmentation does not have a significant impact on the species exchange of *Empoascanara sipra* in the Shibing Yuntai Mountain Reserve so far, with *N_m_* values greater than 1, and species exchanges are more frequent. However, *N_m_* is equal to 0 in the Bijie Salaxi Demonstration Zone and the Zhenfeng-Huajiang Demonstration Zone, indicating that habitat fragmentation has blocked communication between the fragmented populations to a certain extent, and there is no gene communication between the populations in the two study areas.

### 3.4. Correlation Analysis of Erythroneurine Biodiversity and Environmental Factors

#### 3.4.1. Redundancy Analysis (RDA) of Species Diversity of Erythroneurine and Environmental Factors

**Figure 9:**
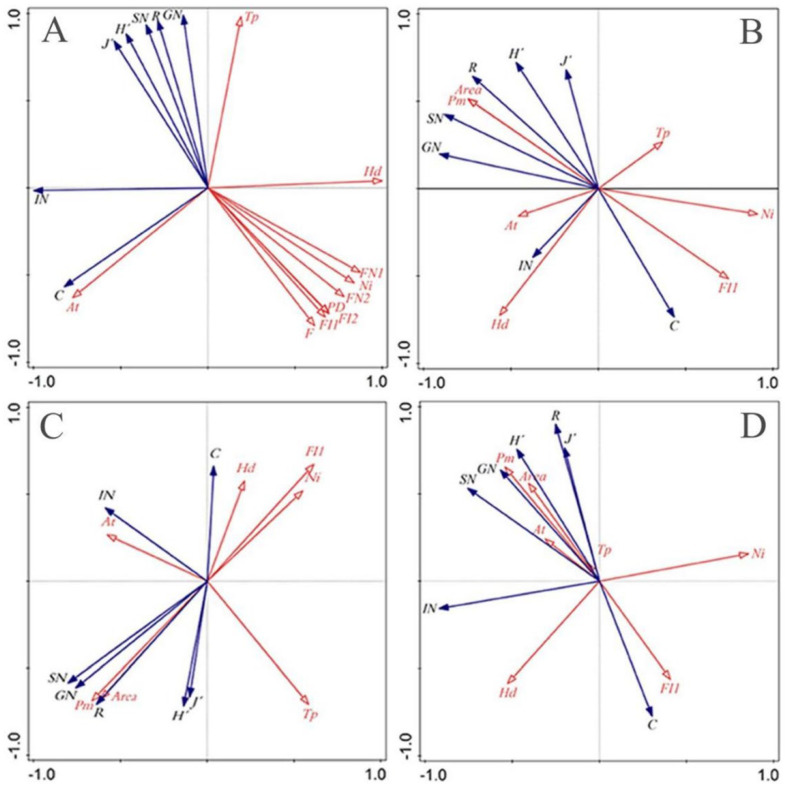
Redundancy analysis of species diversity and geographical environment factors of Erythroneurine leafhoppers in different study areas ((**A**): study area; (**B**): Shibing; (**C**): Bijie; (**D**): Huajiang).

The main environmental factors affecting the occurrence and reproduction of Erythroneurine leafhoppers, such as humidity, temperature, and elevation in the sample plots of the study area, were determined. The specific measurement results are shown in Table S14. Combined with the measured habitat fragmentation data, the biomass data, such as the species diversity index and the number of individuals, genera, and geographic environment factors in the study area, were sorted by RDA. The results are shown in Figure 9.

The first and second axes of RDA (Figure 9A) explain the relationship between the Erythroneurine populations and environmental factors in the study area, which are 92.41% and 7.59%, respectively. It shows that the geographical factors in the area have a great influence on the Erythroneurine population. Temperature *Tp*, humidity *Hd*, altitude *At*, and habitat fragmentation are the main factors affecting the Erythroneurine community. Among them, the *Tp* has a positive correlation with the diversity index (*R*, *H′*, *J′*, *IN*, *GN*) and a negative correlation with the dominance index *C* and the number of individuals *IN*. The relationship between *At* and the Erythroneurine population is opposite to *Tp*; *Hd* and habitat fragmentation index (*PD*, *FN*_1_, *FN*_2_, *Ni*, *F*, *FI*_1_, *FI*_2_) are negatively correlated with species diversity index.

In the Shibing Yuntai Mountain Nature Reserve (Figure 9B), the first and second axes explain 26.41% and 46.51% of all the information, respectively. Habitat fragmentation (*Ni*, *FI*_1_) and *Hd* are the main influencing factors of the Erythroneurine leafhopper community structure and have a negative correlation with related indicators of species diversity. The patch area and the perimeter *Pm* are the opposite, showing a positive correlation, while the effects of other factors are less significant. In the Bijie Salaxi Demonstration Zone (Figure 9C), the first and second axes explain 41.78% and 58.22% of all the information, respectively. Habitat fragmentation (*Ni*, *FI*_1_) and *Tp* are the main factors affecting the distribution of Erythroneurine leafhopper populations and have a negative correlation with species diversity, while the patch area and the perimeter *Pm* show a positive correlation with species diversity. In the Zhenfeng-Huajiang Demonstration Zone (Figure 9D), the first and second axes explain 68.01% and 15.30% of the relationship between Erythroneurine leafhopper populations and environmental factors, respectively. The impacts of seven environmental factors and indicators on the population structure of Erythroneurine leafhoppers from large to small are *Ni* > *Pm* > *Area* > *Hd* > *FI*_1_ > *At* > *Tp*. Among them, habitat fragmentation (*Ni, FI*_1_) and *Hd* are negatively correlated with population species diversity.

Based on the above spatial dynamic analysis, it can be found that the interpretation rates of the first and second axes of the RDA in the three study areas are lower in Shibing, and higher in Bijie and Huajiang, indicating that geographical factors have the least impact on the leafhopper population of Shibing, and which has the greatest impact on the other two regions. In addition to the above geographical factors in the Shibing Yuntai Mountain Nature Reserve, other factors in the area may have a greater impact on the population structure of the Erythroneurine leafhopper, and the relationship between other related factors (such as host plant species, quantity, volatile matter, aspect, light, etc.) and Erythroneurine leafhopper population dynamics needs further study and discussion. However, the population structure in the Bijie Salaxi Demonstration Zone and the Zhenfeng-Huajiang Demonstration Zone are heavily influenced by the above geographical factors. Comparing the RDA results of the study area and each plot, it was found that, in the general environment of the study area, each environmental factor has a significant impact on the leafhopper population, and the relationship between them is consistent with the occurrence of leafhoppers. In the niche with the sample plot as the unit, except for the habitat fragmentation index, the impact of other environmental factors is different and less obvious.

In short, the habitat fragmentation index affects the species diversity in the three study areas and their sample plots and has a negative correlation with it. Among them, the Shibing Yuntai Mountain Nature Reserve has a good ecological environment, with a stable internal ecosystem, and species are less affected by changes in environmental factors. However, the habitat of the Erythroneurine leafhoppers in the other two study areas is relatively harsh, and the species population structure is easily affected by environmental factors.

#### 3.4.2. Redundant Analysis (RDA) of Genetic Diversity of Erythroneurine and Environmental Factors

**Figure 10:**
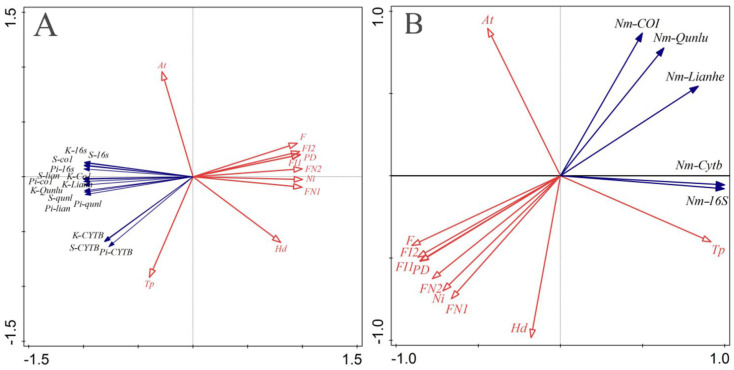
Redundancy analysis of genetic diversity and geographical environment factors of Erythroneurine leafhoppers ((**A**): Genetic diversity parameters; (**B**): Gene−flow).

The first and second axes of RDA (Figure 10) explain the relationships among the genetic diversity parameters, the gene-flow of Erythroneurine populations and the environmental factors in the study area, which are 93.92%, 6.08% and 90.92%, 9.08%, respectively. It shows that the four geographical factors of temperature, humidity, altitude, and habitat fragmentation in the area have a greater impact on the genetic diversity of leafhopper populations, and they are the main influencing factors. Humidity and the habitat fragmentation index are negatively correlated with genetic diversity parameters and gene-flow, while temperature is the opposite and positively correlated with various parameters and gene-flow. Among them, the genetic diversity parameters of *Cytb* are greatly affected by temperature.

#### 3.4.3. Correlation Analysis of Habitat Fragmentation and Erythroneurine Leafhoppers’ Biodiversity

**Figure 11:**
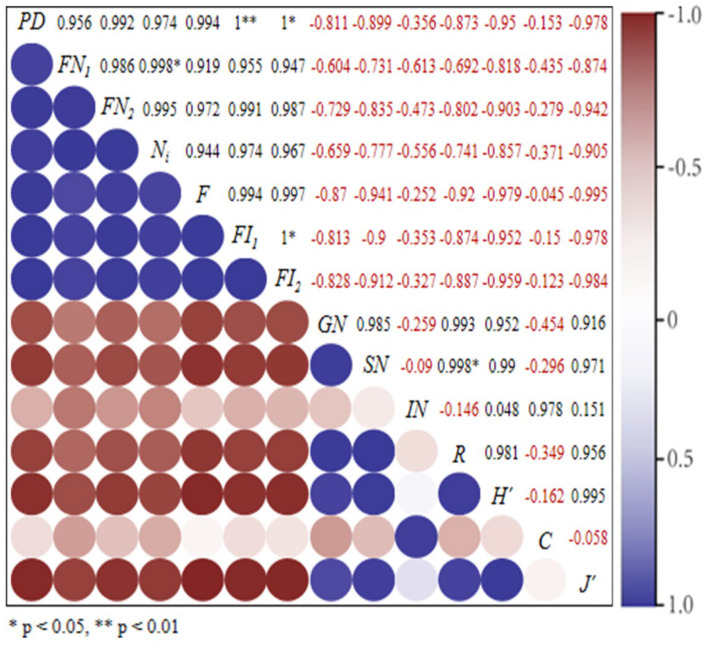
Correlation analysis of Erythroneurine leafhoppers habitat fragmentation and species diversity in the study area.

**Figure 12:**
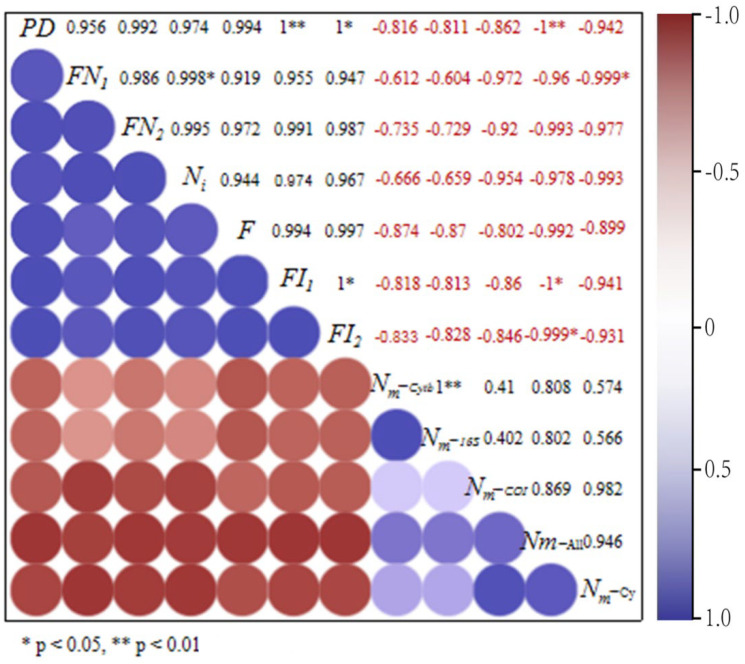
Correlation analysis of Erythroneurine leafhoppers habitat fragmentation and gene−flow.

Based on the above RDA results, a Pearson correlation analysis was used to further verify the relationship between the degree of habitat fragmentation and the biodiversity of Erythroneurine leafhoppers. The study shows (Figure 11 and Figure 12) that the correlation coefficients between the species diversity index and the degree of habitat fragmentation of the leafhoppers are all negative. Except for the number of individuals *IN* and the dominance index *C*, the absolute values of the other indexes are all greater than 0.5, indicating that habitat fragmentation has a significant impact on the diversity of the community structure of leafhoppers in the study area, and there is a negative correlation. In each plot, the correlation coefficients between the species richness index *R*, the diversity index *H′*, the uniformity index *J′*, and the habitat fragmentation index are all negative, and most of the absolute values are around 0.5, indicating that species diversity and habitat fragmentation are negatively correlated, and the relationship between the two is more significant. Instead, the correlation coefficient of the dominance index *C* is positive, being positively correlated with the degree of habitat fragmentation. It shows that the species diversity of Erythroneurine leafhoppers is affected by habitat fragmentation and is negatively related to it. The higher the degree of habitat fragmentation and the lower the uniformity of species distribution, the more prominent the dominant species may be and the lower the diversity of community structure of leafhoppers. In the sample plot, the size of the continuity area of the habitat has a significant impact, and, by affecting the degree of habitat fragmentation, the diversity of fragmented populations is further affected.

For the genetic diversity of Erythroneurine leafhoppers, comparing the correlation coefficients in Figure 12, it can be seen that the correlation coefficients between the genetic diversity, the gene-flow, and the degree of habitat fragmentation among the fragmented populations in the study area are all negative. The absolute values are all greater than 0.5 and close to 1, indicating that habitat fragmentation has a significant impact on species genetic diversity and gene-flow and is negatively correlated. The higher the fragmentation of the habitat of the leafhopper population, the faster the loss of genetic diversity and the greater the barriers to gene exchange between species.

## 4. Discussion

Habitat fragmentation not only affects the migration, spread, establishment, and communication of species, but also affects the integrity of the regional landscape structure and ecosystem, and is one of the most serious threats to plant biodiversity [4]. It is one of the important factors that threaten the survival of natural organisms [51]. This is especially the case in Guizhou, where rocky desertification is more serious, the biological environment is constantly under threat, vegetation coverage is less, and suitable habitats are gradually lost. Habitat fragmentation, as one of the specific results of rocky desertification, is critical to preserving biodiversity and ecosystem function. The community compositions and structures of herbivorous insects are the result of the long-term effects of plants and insects. The density and type of vegetation directly affect the distribution of insect communities, and the diversity of insects and plant diversity are positively correlated [52,53]. Erythroneurine leafhoppers often live on plants and pierce and suck plant sap for food. Their occurrence conditions are closely related to plants, and the composition of the plant community structure in Guizhou karst areas is mostly restricted by the environment. Habitat fragmentation is one of the indicators of biological environmental suitability evaluation, and, like other environmental factors, it has a significant impact on plant diversity. It not only affects the structure of the plant community, but also affects the community structure of related phytophagous insects. The more abundant the habitats of insects, the more diverse the vegetation composition, the higher the community diversity [54,55]; the increase in vegetation abundance and productivity in the area can provide more food resources and living space for Erythroneurine leafhoppers and increase their species diversity [56].

The results of this study are consistent with previous studies, indicating that habitat fragmentation has a significant impact on the number of insect species, community distribution, gene exchange, etc., and is negatively correlated [57,58,59,60,61]. Based on the analysis of the background of the study area, the Shibing Yuntai Mountain Nature Reserve belongs to the karst forest system. The vast majority of the area is natural landscape. The native vegetation is well preserved, and the vegetation coverage is high. Due to the protection policies of the local government, there is little human disturbance, basically no rocky desertification, diverse animal and plant habitats, rich vegetation types, relatively stable internal ecosystems, and strong self-regulation. It can respond to changes in environmental factors in a timely manner and reduce the impact of organisms in the area. It is very suitable for the survival and reproduction of Erythroneurine leafhoppers. In addition, the continuous distribution of forest trees in a large area reduces the degree of habitat fragmentation in the area and provides a foothold for the migration and population exchange process of leafhoppers. The insect biodiversity of this tribe is the highest [62,63]. The Bijie Salaxi Demonstration Area is a typical karst plateau mountainous ecological environment, with peak clusters and depressions in most areas, and the terrain is fragmented. In the early stage, it was severely disturbed by humans, and there were many sloping fields, more damage to the original vegetation, low vegetation coverage, and moderate rocky desertification in most areas [64]. However, in recent years, after returning farmland to forests and other governance measures, the vegetation coverage rate has continued to increase. The vegetation consists mostly of grass and shrub structures [65], and the habitat fragmentation is moderate. Some areas meet the conditions for the occurrence of leafhoppers, but they have a certain blocking effect on the communication between different populations. The Zhenfeng-Huajiang Demonstration Zone belongs to the karst plateau canyon ecological environment, with large undulations, deep valleys, severe damage to native vegetation [66], few plant species, serious soil erosion in the area, high exposure rate of bedrock, and fragile ecosystems, furthermore, the desertification phenomenon is serious [67]. Most areas do not meet the living conditions of leafhoppers, and the number and types of leafhoppers in this area are relatively small.

Based on the above summary and analysis, we have a preliminary understanding of the species diversity and genetic diversity of the Erythroneurine leafhoppers in each study area. The research results show that, as the degree of habitat fragmentation increases, species diversity decreases, and genetic diversity gradually loses. Therefore, in order to better protect the diversity of such insects and their host plants and other biological diversity, combined with the background of the study area, we propose the following reference measures. (1) Strengthen the control of rocky desertification. We can establish a strict forest management system in the region and continue to promote the closure of mountains and forests, conversion of farmland to forests, and grasslands. (2) Enhance farmers’ awareness of ecological protection. Through publicity, the government makes people aware of the importance of ecological protection, reduces deforestation, and helps people establish a stable source of economic income. (3) Increase the continuity of suitable habitats. We can transform rocky desertified land and cultivated land into grassland, shrubland, and woodland and adopt the method of ecological migration to integrate construction land resources. In this way, species relatedness can be improved, and genetic diversity loss reduced. (4) Improve the stability of the ecosystem. We can increase the species diversity of the biological community through artificial introduction and artificial planting, promote the communication between species, and increase the species diversity and genetic diversity of the population. (5) Improve management policies for protected areas. Government departments take the lead based on actual investigations and scientific research analysis results in karst rocky desertification areas to formulate scientific, reasonable, and efficient ecological environment restoration and management protection policies. On the basis of stable restoration, we should explore efficient restoration techniques and methods, and establish a corresponding return-visit evaluation mechanism to effectively improve the efficiency of ecological restoration. 

## 5. Conclusions

In conclusion, this is the first study exploring the genetic diversity and population structure of Erythroneurini in three typical karst areas. The results of the study show that the species diversity and genetic diversity of Erythroneurini leafhoppers in the study area and its various plots are all affected by habitat fragmentation, and there are certain differences in species, quantity, and gene-flow among different fragmented populations. In addition, due to the limited research time, research area, and research populations, although this research reveals some of the problems of biological survival and development in karst areas to a certain extent, the obtained result must be considered as a preliminary exploration based on the scale of habitat fragmentation, and other related issues need to be further explored and studied. In future, we can increase joint research on different scales, combining biomolecules, microorganisms, insects, host plants, the geographical environment on the macro-scale (landscape characteristics) and micro-scale (soil measurement characteristics), biological factors, and non-biological factors to analyze the main influencing factors that affect the formation and distribution of insect community structures. These further studies might reveal the formation mechanism in a comprehensive and thorough analysis. 

## Figures and Tables

**Table 1:** Community structure of Erythroneurine leafhoppers in the study areas.

Genus	Species	Study Areas						Total	Abundance
		SB (1580)		BJ (5245)		HJ (239)			Ra
*Anufrievia*	*Anufrievia* sp. nov.-1	1	+	0		0		1	+
	*Anufrievia parisakazu*	1	+	0		0		1	+
	*Anufrievia* sp.nov.-2	0		2	+	0		2	+
*Arboridia*	*Arboridia* sp. fm-1	1	+	0		0		1	+
	*Arboridia* sp. fm-2	1	+	0		0		1	+
	*Arboridia* *lunula*	16	+	0		0		16	+
	*Arboridia* sp. nov.-1	9	+	0		0		9	+
	*Arboridia* sp. fm-3	0		1	+	0		1	+
	*Arboridia* *echinata*	0		1	+	0		1	+
	*Arboridia* sp. fm-4	0		0		2	+	2	+
*Diomma*	*Diomma pincersa*	1	+	0		0		1	+
*Elbelus*	*Elbelus tripunctatus*	0		0		44	+++	44	+
*Empoascanara*	*Empoascanara* sp. fm-1	1	+	0		0		1	+
	*Empoascanara* sp. fm-2	1	+	0		0		1	+
	*Empoascanara* sp. fm-3	1	+	0		0		1	+
	*Empoascanara* sp. fm-4	1	+	0		0		1	+
	*Empoascanara* sp. fm-5	0		2	+	0		2	+
	*Empoascanara sipra*	1267	+++	5185	+++	13	+++	6465	+++
	*Empoascanara dwalata*	4	+	0		0		4	+
	*Empoascanara gracilis*	9	+	0		0		9	+
	*Empoascanara* sp. fm-6	0		0		4	++	4	+
	*Empoascanara mai*	0		0		1	+	1	+
	*Empoascanara* sp. nov.-1	0		0		1	+	1	+
*Kapsa*	*Kapsa* sp. fm-1	1	+	0		0		1	+
	*Kapsa alba*	0		3	+	0		3	+
	*Kapsa arca*	0		1	+	0		1	+
	*Kapsa dolka*	0		12	+	0		12	+
	*Kapsa* sp. nov.-1	0		0		5	++	5	+
	*Kapsa dolka*	0		0		2	+	2	+
*Mitjaevia*	*Mitjaevia* *dworakowskae*	33	++	0		0		33	+
	*Mitjaevia aurantiaca*	6	+	0		0		6	+
	*Mitjaevia* sp. fm-1	1	+	0		0		1	+
	*Mitjaevia* sp. fm-2	2	+	0		0		2	+
	*Mitjaevia shibingensis*	69	++	0		0		69	+
	*Mitjaevia protuberanta*	19	++	0		0		19	+
	*Mitjaevia diana*	0		12	+	0		12	+
	*Mitjaevia* sp. nov.-1	0		15	+	0		15	+
	*Mitjaevia* sp. nov.-2	0		1	+	0		1	+
	*Mitjaevia* sp. nov.-3	0		1	+	0		1	+
	*Mitjaevia* sp. fm-3	0		3	+	0		3	+
	*Mitjaevia* sp. fm-4	0		0		1	+	1	+
*Salka*	*Salka sawna*	19	++	4	+	0		23	+
*Seriana*	*Seriana bacilla*	100	++	0		142	+++	242	++
	*Seriana ochrata*	0		0		12	+++	12	+
*Tautoneura*	*Tautoneura albida*	0		1	+	0		1	+
*Thaia*	*Thaia* sp. fm-1	0		1	+	0		1	+
	*Thaia* sp. fm-2	0		0		3	++	3	+
	*Thaia* sp. fm-3	0		0		1	+	1	+
*Ziczacella*	*Ziczacella steggerdai*	7	+	0		0		7	+
Erythroneurini new-1	Erythroneurini Gen. new-1. sp. nov.	9	+	0		0		9	+
Erythroneurini new-2	Erythroneurini Gen. new-2. sp. nov.	0		0		8	++	8	+

Note: +: rare taxa is <1%, ++: common taxa is 1~5%, +++: dominant taxa is >5%; SB: Shibing Yuntai Mountain, BJ: Bijie Salaxi Demonstration Zone, HJ: Zhenfeng-Huajiang Demonstration Zone, sp. nov: an unidentified species in this genus, sp. fm: a female species not identified in the genus.

**Table 2:** Nucleotide polymorphism parameters of Erythroneurine leafhoppers population in different study areas.

Genes	Study Areas	Length bp	Nucleotide Diversity *P_i_*	Average Number of Nucleotide Differences *K*	Number of Polymorphic (Segregating) Sites *S*	Haplotypes *H*
*Cytb*	SB	659	0.0111	7.333	11	3
	BJ	641	0.0052	3.333	5	3
	HJ	630	0.0042	2.667	4	3
*16S*	SB	493	0.0095	4.667	7	3
	BJ	486	0.0082	4.000	6	2
	HJ	476	0.0014	0.667	1	2
*COI*	SB	683	0.0059	4.000	6	3
	BJ	714	0.0047	3.333	5	2
	HJ	683	0.0010	0.667	1	2
*Cytb +* *16S + COI*	SB	1835	0.0288	16.008	24	3
	BJ	1841	0.0192	10.672	16	3
	HJ	1789	0.0072	4.002	6	3

## Data Availability

All data generated or analyzed in this study are included in this published article and its supplementary information files.

## Supplementary Materials

The following supporting information can be downloaded at: https://www.mdpi.com/article/10.3390/insects13060499/s1, Table S1: Overview of sample plots. Table S2: Sequencing species information. Table S3: Primer names, sequences used in PCR reactions of genes sequenced. Table S4: The reaction system for PCR. Table S5: The thermal cycling condition for gene. Table S6: Habitat fragmentation index of Erythroneurine leafhoppers in three different study areas. Table S7: Habitat fragmentation index of Erythroneurine leafhoppers in the sample plots. Table S8: Species diversity of leafhoppers in different sample plots of Shibing. Table S9: Species diversity of leafhoppers in different sample plots of Bijie. Table S10: Species diversity of leafhoppers in different sample plots of Huajiang. Table S11: Community diversity of Erythroneurine leafhoppers in different study areas. Table S12: Community diversity of Erythroneurine leafhoppers in each sample plot. Table S13: Gammast and Fst values of fragmented populations of Erythroneurine leafhoppers in the study area. Table S14: Measured values of environmental factors in different study areas and plots.
