# Supplementary material for: Effects of Habitat Fragmentation on the Population Structure and Genetic Diversity of Erythroneurini in the Typical Karst Rocky Ecosystem, Southwest China
Source: Insects. 2022 May 26;13(6):499. doi: 10.3390/insects13060499 (PMC9225061; doi:10.3390/insects13060499)
Supplement: Supplementary file 1 [file insects-13-00499-s001.zip › insects-1723073-supplementary.pdf]

Table S1 Overview of sample plots

Study areas	Sample plots	Vegetation	Humidity	
Shibing	SB1	grass	27°9′50″ N	108°9′14″ E
	SB2	grass	27°9′9″ N	108°7′20″ E
	SB3	grass	27°12′27″ N	108°2′38″ E
	SB4	grass	27°8′56″ N	108°1′51″ E
	SB5	grass	27°5′21″ N	108°4′30″ E
	SB6	Grass and trees	27°8′21″ N	108°2′18″ E
	SB7	Grass and shrubs	27°13′28″ N	108°1′34″ E
	SB8	grass	27°9′32″ N	108°9′42″ E
	SB9	Shrubs and trees	27°9′59″ N	108°4′19″ E
Bijie	BJ1	grass	27°14′27″ N	105°8′54″ E
	BJ2	Grass and shrubs	27°15′1″ N	105°6′32″ E
	BJ3	Grass and shrubs	27°15′33″ N	105°6′49″ E
	BJ4	grass	27°13′37″ N	105°3′23″ E
	BJ5	grass	27°12′25″ N	105°4′51″ E
	BJ6	grass	27°14′2″ N	105°3′51″ E
	BJ7	grass	27°16′21″ N	105°4′40″ E
	BJ8	Shrubs and trees	27°14′7″ N	105°6′26″ E
	BJ9	grass	27°12′36″ N	105°7′35″ E
Huajiang	HJ1	grass	25°40′38″ N	105°41′5″ E
	HJ2	grass	25°38′27″ N	105°39′18″ E
	HJ3	Shrubs and trees	25°39′26″ N	105°39′0″ E
	HJ4	grass	25°38′59″ N	105°38′6″ E
	HJ5	Shrubs and trees	25°39′11″ N	105°37′46″ E
	HJ6	grass	25°38′49″ N	105°38′17″ E
	HJ7	grass	25°41′10″ N	105°37′14″ E
	HJ8	Shrubs and trees	25°39′38″ N	105°38′50″ E
	HJ9	grass	25°39′17″ N	105°37′33″ E

Table S2 Sequencing species information

Study area	Sample plots	Habitat fragmentation degree	Species	Sequencing genes
Shibing	SB1	low	*E. sipra*	*Cytb/16S/COI*
	SB7	medium	*E. sipra*	*Cytb/16S/COI*
	SB4	high	*E. sipra*	*Cytb/16S/COI*
Bijie	BJ9	low	*E. sipra*	*Cytb/16S/COI*
	BJ5	medium	*E. sipra*	*Cytb/16S/COI*
	BJ2	high	*E. sipra*	*Cytb/16S/COI*
Huajiang	HJ1	low	*E. sipra*	*Cytb/16S/COI*
	HJ8	medium	*E. sipra*	*Cytb/16S/COI*
	HJ5	high	*E. sipra*	*Cytb/16S/COI*

Note: The degree of habitat fragmentation is compared to the degree of fragmentation of the sample plots in each study area.

Table S3 Primer names, sequences used in PCR reactions of genes sequenced

Gene	Primer name	Primer sequence 5'–3'	Reference
COI	LCO1490	GGTCAACAAATCATAAAGATATTGG	Simon *et al*. (1994)
	HCO2198	TAAACTTCAGGGTGACCAAAAAATCA	
Cytb	CytbF	GTTCTACCTTGAGGTCAAATATC	Simon *et al*. (1994)
	CytbR	TTCTACTGGTCGTGCTCCAATTCA	
16S	LR-J-12887	CCG GTY TGA ACT CAR ATC AWG T	Dietrich *et al*. (1997)
	LR-N-13398	CTG TTT AWC AAA AAC ATT TC	

Table S4 The reaction system for PCR

Reagent	Volume
CWBIO 2×Es Taq Master Mix	12.5
dd H2O	8.5
DNA template	2
Upstream primer（10 ul/L）	1
Downstream primer（10 ul/L）	1

Table S5 The thermal cycling condition for *COI* gene

Amplification program	Time
94°C pre-denaturation	2.0 min
94°C denaturation	30 s
51.4°C annealing	1.0 min, 35 cycles
72°C extension	50 s
72°C final extension	10.0 min
4°C constant temperature	Forever

Table S6 Habitat fragmentation index of Erythroneurine leafhoppers in three different study areas

Study areas	Patchdensity index	Landscape fragmentation index		Separateness index	Fragmentation index	Inside habitatarea fragmentation index	
	PD	FN_1	FN_2	N_i	*F*	FI_1	FI_2
Shibing	0.1223	0.0110	0.0015	0.0004	0.0075	0.0894	0.0975
Bijie	0.2304	0.0142	0.0066	0.0012	0.3705	0.3195	0.4261
Huajiang	0.3850	0.0346	0.0187	0.0043	0.7246	0.6447	0.8477

Table S7 Habitat fragmentation index of Erythroneurine leafhoppers in the sample plots

Study areas	Sample plots	Patch area hm^2	Patch perimeter km	Separateness index N_i	Inside habitatarea fragmentation index FI_1
Shibing	SB1	22910.09	908.42	0.0000	0.0089
	SB2	22910.09	908.42	0.0000	0.0089
	SB3	22910.09	908.42	0.0000	0.0089
	SB4	2.38	0.81	0.3196	0.9999
	SB5	22910.09	908.42	0.0000	0.0089
	SB6	18.67	2.81	0.0407	0.9992
	SB7	60.85	7.26	0.0125	0.9974
	SB8	5.94	1.36	0.1280	0.9997
	SB9	22910.09	908.42	0.0000	0.0089
Bijie	BJ1	2.26	0.90	0.1888	0.9997
	BJ2	95.89	17.27	0.0044	0.9868
	BJ3	1.05	0.44	0.4078	0.9999
	BJ4	12.02	3.37	0.0355	0.9983
	BJ5	21.36	4.82	0.0200	0.9971
	BJ6	206.95	29.84	0.0021	0.9715
	BJ7	19.59	4.35	0.0218	0.9973
	BJ8	106.47	18.65	0.0040	0.9854
	BJ9	108.14	21.00	0.0039	0.9851
Huajiang	HJ1	24.02	9.30	0.0090	0.9871
	HJ2	7.38	2.82	0.0293	0.9960
	HJ3	10.43	3.30	0.0207	0.9944
	HJ4	23.85	4.10	0.0091	0.9872
	HJ5	5.94	2.16	0.0364	0.9968
	HJ6	2.07	0.79	0.1045	0.9989
	HJ7	108.34	9.08	0.0020	0.9419
	HJ8	13.50	2.64	0.0160	0.9928
	HJ9	0.86	0.46	0.2500	0.9995

Table S8 Species diversity of leafhoppers in different sample plots of Shibing

Sample plots	Genus	Species	Number of individuals	Abundance
SB1	*Arboridia*	*Arboridia lunula*	2	++
		Arboridia sp.fm-2	1	+
		Arboridia sp.nov-1	6	+++
	Empoascanara	*Empoascanara sipra*	61	+++
	Mitjaevia	*Mitjaevia* sp.fm-2	2	++
		Mitjaevia protuberanta	12	+++
	Salka	*Salka sawna*	6	+++
	Seriana	*Seriana bacilla*	17	+++
SB2	*Arboridia*	*Arboridia lunula*	7	+++
	Diomma	*Diomma (Diomma) pincersa*	1	++
	Empoascanara	*Empoascanara sipra*	14	+++

	Mitjaevia	*Mitjaevia aurantiaca*	4	+++
		Mitjaevia sp.fm-1	1	++
		Mitjaevia protuberanta	4	+++
	Salka	*Salka sawna*	1	++
	Seriana	*Seriana bacilla*	10	+++
	Erythroneurini new-1	Erythroneurini new-1.sp.nov.	3	+++
SB3	*Arboridia*	*Arboridia* sp.fm-1	1	+
	*Anufrievi*a	*Anufrievi*a parisakazu	1	+
	Empoascanara	*Empoascanara sipra*	417	+++
		Empoascanara sp.fm-2	1	+
	Salka	*Salka sawna*	1	+
	Seriana	*Seriana bacilla*	1	+
	Erythroneurini new-1	Erythroneurini new-1.sp.nov.	3	+
	Ziczacella	*Ziczacella steggerdai*	6	++
SB4	*Empoascanara*	*Empoascanara sipra*	100	+++
	Seriana	*Seriana bacilla*	5	++
SB5	*Arboridia*	*Arboridia lunula*	6	+++
	Empoascanara	*Empoascanara dwalata*	4	++
		Empoascanara sipra	29	+++
		Empoascanara gracilis	9	+++
	Kapsa	*Kapsa* sp.fm-1	1	++
	Salka	*Salka sawna*	5	+++
	Seriana	*Seriana bacilla*	25	+++
	Erythroneurini new-1	Erythroneurini new-1.sp.nov.	2	++
SB6	*Arboridia*	*Arboridia lunula*	1	+
	Empoascanara	*Empoascanara sipra*	203	+++
	Mitjaevia	*Mitjaevia aurantiaca*	1	+
	Salka	*Salka sawna*	3	++
	Seriana	*Seriana bacilla*	17	+++
SB7	*Empoascanara*	*Empoascanara sipra*	433	+++
	Mitjaevia	*Mitjaevia protuberanta*	1	+
	Salka	*Salka sawna*	1	+
	Seriana	*Seriana bacilla*	7	++
	Ziczacella	*Ziczacella steggerdai*	1	+
SB8	*Empoascanara*	*Empoascanara sipra*	6	+++
	Mitjaevia	*Mitjaevia aurantiaca*	1	+++
	Erythroneurini new-1	Erythroneurini new-1.sp.nov.	1	+++
SB9	*Arboridia*	*Arboridia* sp.nov-1	3	++
	Anufrievia	*Anufrievia* sp.nov-1	1	+
	Empoascanara	*Empoascanara sipra*	4	++
		Empoascanara sp.fm-1	1	+
		Empoascanara sp.fm-3	1	+
		Empoascanara sp.fm-4	1	+
	Mitjaevia	*Mitjaevia dworakowskae*	33	+++

		Mitjaevia shibingensis	69	+++
		Mitjaevia protuberanta	2	++
	Salka	*Salka sawna*	3	++
	Seriana	*Seriana bacilla*	17	+++

Table S9 Species diversity of leafhoppers in different sample plots of Bijie

Sample plots	Genus	Species	Number of individuals	Abundance
BJ1	*Empoascanara*	*Empoascanara sipra*	107	+++
BJ2	*Empoascanara*	*Empoascanara sipra*	1727	+++
		Empoascanara sp.fm-5	2	+
	Kapsa	*Kapsa dolka*	1	+
		Kapsa alba	1	+
	Mitjaevia	*Mitjaevia diana*	1	+
	Salka	*Salka sawna*	2	+
BJ3	*Empoascanara*	*Empoascanara sipra*	459	+++
BJ4	*Empoascanara*	*Empoascanara sipra*	795	+++
	Mitjaevia	*Mitjaevia* sp.fm-3	1	+
BJ5	*Empoascanara*	*Empoascanara sipra*	473	+++
	Mitjaevia	*Mitjaevia* sp.nov-1	1	+
		Mitjaevia diana	3	+
BJ6	*Empoascanara*	*Empoascanara sipra*	448	+++
	Kapsa	*Kapsa dolka*	3	+
	Mitjaevia	*Mitjaevia* sp.nov-1	11	++
		Mitjaevia sp.nov-2	1	+
		Mitjaevia sp.nov-3	1	+
		Mitjaevia sp.fm-3	2	+
		Mitjaevia diana	1	+
	Salka	*Salka sawna*	1	+
	Thaia	*Thaia* sp.fm-1	1	+
BJ7	*Empoascanara*	*Empoascanara sipra*	288	+++
	Kapsa	*Kapsa dolka*	1	+
	Mitjaevia	*Mitjaevia* sp.nov-1	1	+
BJ8	*Arboridia*	*Arboridia echinata*	1	++
	Empoascanara	*Empoascanara sipra*	31	+++
	Kapsa	*Kapsa arca*	1	++
	Mitjaevia	*Mitjaevia* sp.nov-1	2	+++
		Mitjaevia diana	2	+++
	Salka	*Salka sawna*	1	++
BJ9	*Arboridia*	*Arboridia* sp.fm-3	1	+
	Anufrievia	*Anufrievia* sp.nov-2	2	+
	Empoascanara	*Empoascanara sipra*	857	+++
	Kapsa	*Kapsa dolka*	7	+
		Kapsa alba	2	+
	Mitjaevia	*Mitjaevia diana*	5	+

	Tautoneura	*Tautoneura albida*	1	+

Table S10 Species diversity of leafhoppers in different sample plots of Huajiang

Sample plots	Genus	Species	Number of individuals	Abundance
HJ1	*Arboridia*	*Arboridia* sp.fm-4	1	+++
	Empoascanara	*Empoascanara sipra*	2	+++
	Kapsa	*Kapsa dolka*	1	+++
	Seriana	*Seriana bacilla*	9	+++
	Thaia	*Thaia* sp.fm-2	3	+++
HJ2	*Empoascanara*	*Empoascanara* sp.fm-6	2	++
		Empoascanara sipra	2	++
	Seriana	*Seriana bacilla*	45	+++
HJ3	*Elbelus*	*Elbelus tripunctatus*	21	+++
	Empoascanara	*Empoascanara* sp.fm-6	1	++
		Empoascanara sipra	3	+++
HJ4	*Arboridia*	*Arboridia* sp.fm-4	1	++
	Empoascanara	*Empoascanara mai*	1	++
	Seriana	*Seriana ochrata*	8	+++
		Seriana bacilla	47	+++
	Thaia	*Thaia* sp.fm-3	1	++
HJ5	*Empoascanara*	*Empoascanara sipra*	1	+++
	Seriana	*Seriana bacilla*	8	+++
HJ6	*Seriana*	*Seriana bacilla*	3	+++
HJ7	Erythroneurini new-2	Erythroneurini new-2.sp.nov.	8	+++
	Empoascanara	*Empoascanara* sp.fm-6	1	++
		Empoascanara sipra	3	+++
	Kapsa	*Kapsa* sp.nov-1	5	+++
		Kapsa dolka	1	++
	Seriana	*Seriana bacilla*	7	+++
HJ8	*Elbelus*	*Elbelus tripunctatus*	23	+++
	Empoascanara	*Empoascanara sipra*	2	++
	Seriana	*Seriana ochrata*	4	+++
		Seriana bacilla	23	+++
HJ9	*Empoascanara*	*Empoascanara* sp.nov-1	1	+++
	Mitjaevia	*Mitjaevia* sp.fm-4	1	+++

Table S11 Community diversity of Erythroneurine leafhoppers in different study areas

Study areas	Number of genus *GN*	Number of species *SN*	Number of individuals *IN*	Mrrgalef Species richness index *R*	Shannon-Wiener Diversity index *H´*	BSimpson Dominance index *C*	Pieluo Uniformity index *J´*
Shibing	10	25	1580	3.2719	0.5594	0.0318	0.1645
Bijie	8	16	5245	1.6924	0.2862	0.5382	0.1032

Huajiang	8	14	239	1.4667	0.1625	0.0005	0.0616

Table S12 Community diversity of Erythroneurine leafhoppers in each sample plot

Study areas	Sample plots	Number of genus *GN*	Number of species *SN*	Number of individuals *IN*	Mrrgalef Species richness index *R*	Shannon-Wiener Diversity index *H´*	BSimpson Dominance index *C*	Pieluo Uniformity index *J´*
Shibing	SB1	5	8	107	1.4980	1.3736	0.3699	0.6606
	SB2	7	9	45	2.1016	1.9831	0.1687	0.9025
	SB3	7	9	431	1.3188	0.3307	0.8798	0.1505
	SB4	2	2	105	0.2149	0.1914	0.9093	0.2761
	SB5	6	8	81	1.5929	1.6336	0.2483	0.7856
	SB6	5	5	225	0.7385	0.3937	0.8199	0.2446
	SB7	5	5	443	0.6564	0.1291	0.9556	0.0802
	SB8	3	3	8	0.9618	0.7356	0.5938	0.6696
	SB9	6	11	135	2.0386	1.4295	0.3391	0.5961
Bijie	BJ1	1	1	107	0.0000	0.0000	1.0000	0.0000
	BJ2	4	6	1734	0.6704	0.0325	0.9919	0.0182
	BJ3	1	1	459	0.0000	0.0000	1.0000	0.0000
	BJ4	2	2	796	0.1497	0.0096	0.9975	0.0139
	BJ5	2	3	477	0.3243	0.0532	0.9833	0.0484
	BJ6	5	9	469	1.3007	0.2529	0.9131	0.1151
	BJ7	3	3	290	0.3527	0.0460	0.9863	0.0418
	BJ8	5	6	38	1.3745	0.7632	0.6731	0.4260
	BJ9	6	7	875	0.8857	0.1318	0.9594	0.0677
Huajiang	HJ1	5	5	16	1.4427	1.2440	0.3750	0.7730
	HJ2	2	3	49	0.5139	0.3393	0.8467	0.3089
	HJ3	2	3	25	0.6213	0.5296	0.7216	0.4821
	HJ4	4	5	58	0.9851	0.6537	0.6766	0.4062
	HJ5	2	2	9	0.4551	0.3488	0.8025	0.5033
	HJ6	1	1	3	0.0000	0.0000	1.0000	0.0000
	HJ7	4	6	25	1.5533	1.5549	0.2384	0.8678
	HJ8	3	4	52	0.7593	1.0442	0.3987	0.7533
	HJ9	2	2	2	1.4427	0.6931	0.5000	1.0000

Table S13 $Gamma_{st}$ and F_{st} values of fragmented populations of Erythroneurine leafhoppers in the study area

Gene	Study area	Population	1	2	3
Cytb	Shibing	1	—	-0.9091	-0.0667
		2	0.0233	—	-0.6250
		3	0.0278	0.0365	—
	Bijie	1	—	-0.4000	-0.6000
		2	0.1765	—	0.0000
		3	0.1111	0.3333	—
	Huajiang	1	—	-0.2500	0.0000

Gene	Study area	Population	1	2	3
		2	0.2308	—	-0.7500
		3	0.3333	0.1067	—
16S rRNA	Shibing	1	—	-0.5714	-0.4286
		2	0.1200	—	0.0000
		3	0.1667	0.3333	—
	Bijie	1	—	-1.0000	0.0000
		2	0.0000	—	0.0000
		3	0.3333	0.3333	—
	Huajiang	1	—	0.0000	0.0000
		2	0.3333	—	-1.0000
		3	0.3333	0.0000	—
COI	Shibing	1	—	-0.1667	-0.5000
		2	0.2632	—	-0.3333
		3	0.1429	0.2000	—
	Bijie	1	—	-0.2000	-0.6000
		2	0.2500	—	-0.2000
		3	0.1111	0.2500	—
	Huajiang	1	—	-1.0000	0.0000
		2	0.0000	—	0.0000
		3	0.3333	0.3333	—
Combine	Shibing	1	—	-0.6250	-0.2917
		2	0.1035	—	-0.0833
		3	0.2152	0.2973	—
	Bijie	1	—	-0.5625	-0.3750
		2	0.1228	—	-0.0625
		3	0.1852	0.3061	—
	Huajiang	1	—	-0.3333	0.0000
		2	0.2000	—	-0.6667
		3	0.3333	0.1333	—

Note: Below the diagonal: $Gamma_{st}$ values, above the diagonal: F_{st} values.

Table S14 Measured values of environmental factors in different study areas and plots

Study areas	Sample plots	Humidity(%)	Temperature(℃)	Altitude(m)
Shibing	—	64	27	927
Bijie	—	57	24	1731
Huajiang	—	74	25	941
Shibing	SB1	64	29	1034
	SB2	57	27.5	969
	SB3	71	24.5	1187
	SB4	60	30.5	575
	SB5	72.5	28	702
	SB6	68	28.5	896.5
	SB7	63	25.5	1059
	SB8	56.5	26.5	996
	SB9	54.5	28.5	855
Bijie	BJ1	59.5	28	1279.5

	BJ2	63	24	1879
	BJ3	67.5	23.5	1825
	BJ4	60.5	24.8	1862.5
	BJ5	49.5	22.5	1537
	BJ6	54.5	25.5	1785.5
	BJ7	67	24	1942
	BJ8	53.5	26.5	1677
	BJ9	49.5	24.5	1643
Huajiang	HJ1	75	26	999.5
	HJ2	77	25	1084
	HJ3	79	20.5	762
	HJ4	71	28	935
	HJ5	71	28	935
	HJ6	75	27	890
	HJ7	68.2	29.8	1078
	HJ8	81	22	864
	HJ9	60	22	882
